# Study of Different Variants of Mo Enzyme crARC and the Interaction with Its Partners crCytb5-R and crCytb5-1

**DOI:** 10.3390/ijms18030670

**Published:** 2017-03-21

**Authors:** Alejandro Chamizo-Ampudia, Aurora Galvan, Emilio Fernandez, Angel Llamas

**Affiliations:** Departamento de Bioquímica y Biología Molecular, Universidad de Córdoba, Campus de Rabanales, Edificio Severo Ochoa, 14071 Córdoba, Spain; b42chaaa@uco.es (A.C.-A.); bb1gacea@uco.es (A.G.); bb1feree@uco.es (E.F.)

**Keywords:** mARC, Chlamydomonas, amidoxime, HAP, molybdenum, partners, interaction, oligomers

## Abstract

The mARC (mitochondrial Amidoxime Reducing Component) proteins are recently discovered molybdenum (Mo) Cofactor containing enzymes. They are involved in the reduction of several *N*-hydroxylated compounds (NHC) and nitrite. Some NHC are prodrugs containing an amidoxime structure or mutagens such as 6-hydroxylaminopurine (HAP). We have studied this protein in the green alga *Chlamydomonas reinhardtii* (crARC). Interestingly, all the ARC proteins need the reducing power supplied by other proteins. It is known that crARC requires a cytochrome *b*_5_ (crCytb5-1) and a cytochrome *b*_5_ reductase (crCytb5-R) that form an electron transport chain from NADH to the substrates. Here, we have investigated NHC reduction by crARC, the interaction with its partners and the function of important conserved amino acids. Interactions among crARC, crCytb5-1 and crCytb5-R have been studied by size-exclusion chromatography. A protein complex between crARC, crCytb5-1 and crCytb5-R was identified. Twelve conserved crARC amino acids have been substituted by alanine by in vitro mutagenesis. We have determined that the amino acids D182, F210 and R276 are essential for NHC reduction activity, R276 is important and F210 is critical for the Mo Cofactor chelation. Finally, the crARC C-termini were shown to be involved in protein aggregation or oligomerization.

## 1. Introduction

**Figure 1:**
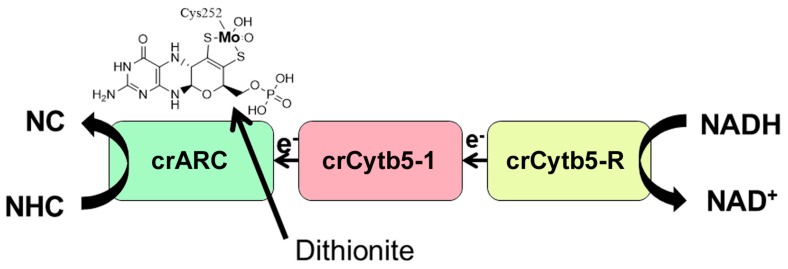
Scheme of NHC reduction to N-compounds (NC) by the Chlamydomonas ARCO. Each protein involved is represented schematically in boxes. The crARC protein is able to bind Mo Cofactor, which is indicated by the Mo Cofactor chemical structure above the protein box. In bold, the Mo atom, bound to the organic motif MPT, illustrating that the fifth Mo ligand in crARC is the cysteine 252. In vitro, the NHC reduction (HAP or benzamidoxime) mediated by crARC to NC (adenine or benzamidine, respectively) could occur in two ways, using dithionite or NADH as electron donors. Dithionite is an artificial electron donor that directly donates the electrons to the crARC Mo centre which is indicated by the arrow. The NADH donates the electrons to crCytb5-R, then to crCytb5-1, and then to the crARC Mo centre where NHC reduction takes place.

Mo is the only second-row transition metal that participates in critical biological functions in most living beings from bacteria to humans [1]. For gaining biological activity and fulfilling its function in enzymes, all studied eukaryotic Mo enzymes have Mo chelated by a tricyclic pyranopterin compound called molybdopterin (MPT), thus forming the Mo Cofactor [2] (Figure 1). The Mo Cofactor is widespread in all kingdoms and synthesized by a very conserved pathway [3]. The Mo Cofactor takes part in the active centre of more than fifty different enzymes involved in key reactions of nitrogen and sulphur metabolism, phytohormone biosynthesis and detoxification of xenobiotics. Most of these Mo-containing enzymes occur in prokaryotes while only five of them have been identified in eukaryotes: nitrate reductase (NR), sulphite oxidase (SO), aldehyde oxidase (AO), xanthine oxidoreductase (XOR), and mARC the last one uncovered [4].

mARC proteins are members of the MOSC protein superfamily [5]. These proteins contain a domain homologous to the C-terminal domain (MOSC) that is also present in the eukaryotic Mo Cofactor Sulphurases (MOS). The MOS enzymes are involved in the transfer of a sulfide ligand yielding a sulfurated version of the Mo Cofactor which is essential for the activity of xanthine oxidase (XO) family of Mo Cofactor enzymes [1]. However except for the MOS, all other members of the MOSC superfamily, including mARC, are proteins without any confirmed physiological function.

Investigation of the aerobic reduction of NHC led to the discovery of an unknown Mo enzyme [4]. The protein was named “mitochondrial amidoxime reducing component” (mARC), because initially, reduction of amidoxime structures was studied with this enzyme purified from porcine liver mitochondria. Afterwards, it was discovered that mARC proteins are also widely distributed and involved in reducing a broad range of other NHC [6]. It is well accepted that the human enzyme system contributes to reductive xenobiotic metabolism, in particular, activation of *N*-hydroxylated prodrugs [7]. Further substrates are the base analogs HAP [8] and the *N*-hydroxy-cytosine [9,10], which are very powerful mutagens in phage, bacteria and eukaryotic cells [11]. In bacteria, the defect in any enzyme involved in the Mo Cofactor biosynthesis pathway gives a HAP-hypersensitive phenotype, the first evidence seen in an Mo Cofactor dependent enzyme involved in the detoxification of HAP [12].

Interestingly, the mARC enzymes need additional proteins which act as their partners and donate the reducing power necessary for the substrate reductions. Therefore, we proposed that the complex formed between mARC and their partners can be called ARCO (**A**midoxime **R**educing **CO**mplex) [13]. Humans, similar to many eukaryotes, contain two mARC versions (hmARC1 and hmARC2), which both show strong similarities at amino acid and nucleotide levels. In the presence of NADH, each human Mo enzyme exerts reductase activity towards NHCs with cytochrome *b*_5_ [14] and NADH cytochrome *b*_5_ reductase [15]. However, the bacterial ARCO is a two-component system that uses ferredoxin fused to the C-termini mARC enzyme (YcbX) instead of cytochrome and as a second component, uses flavin reductase (CysJ) instead of NADH cytochrome *b*_5_ reductase [16].

The first ARCO studied in detail in the plant kingdom was in the alga (*Chlamydomonas reinhardtii*) (Chlamydomonas). We found that Chlamydomonas ARCO is the Mo enzyme crARC, the cytochrome *b*_5-1_ (crCytb5-1) and one NADH cytochrome *b*_5_ reductase (crCytb5-R) (Figure 1), similar to its human counterpart and different from the two components in a prokaryotic system [17]. The Chlamydomonas ARCO is able to reduce HAP to adenine, shows a Zn^2+^-dependent activity that increases its *V*_max_ more than 14-fold, and belongs to the SO family since its Cysteine 252 was identified as a putative ligand of the Mo atom [17] (Figure 1).

At present, the physiological role of ARCO is a matter of intense debate [7]. In addition to NHC, mARC is able to reduce other kinds of substrates, like nitrite [18]; however, using different kinds of partners for each of them, like NR [19]. Recently, apart from NHC reduction, the mARC proteins have been shwon to be involved in NO synthesis by different ways. In this regard, in vitro studies have shown that human ARCO is also able to catalyze the reduction of the NO precursor *N*-omega-hydroxy-l-arginine (NOHA) to arginine [20]. In this sense, a variety of several enzymes have been involved with in vitro synthesis of NO by reducing nitrite, and Mo enzymes like NR, SO and XOR appear in many of them [21]. The human ARCO apart from the NHC and NOHA reductions is also able to catalyze the reduction of nitrite to NO using cytochrome *b*_5*-1*_ and cytochrome *b*_5_ reductase as partners [18], however, only under anaerobic conditions.

Interestingly, in the process of NO synthesis in Chlamydomonas, the NR, another Mo enzyme, is able to replace very efficiently in ARCO the function of crCytb5-R and crCytb5-1 and reduce nitrite to NO [19]. Therefore, this new mARC activity able to synthesize NO but NR-dependent has been renamed as NOFNiR (**N**itric **O**xide **F**orming **Ni**trite **R**eductase) [19]. Here, we have not addressed the NOFNiR dual system crARC-NR involved in the NO synthesis; rather, we have focused on the NHC reduction catalyzed by crARC, crCytb5-1 and crCytb5-R. Also, we have described the crARC stability at different temperatures, the stoichiometry of the interaction between crARC and its partners crCytb5-1 and crCytb5-R, and the function of different crARC conserved amino acids in NHC reduction activity.

## 2. Results

### 2.1. The Temporal Stability of the crARC Activity

There is still no report of the behavior of any mARC proteins over the time of the reduction activity. It is known that the activity under aerobic conditions of most Mo Cofactor proteins is very low, and usually after a few hours they start to lose their activity [22]. Accordingly, we have studied the stability of crARC activity at 22 and 4 °C by determining its NHC reduction activity at different times.

The NHC substrate selected to be reduced in this assay was the model substrate benzamidoxime [4]. The crARC reduction activity was determined in vitro in two ways, using either dithionite or NADH as electron donors. Dithionite is an artificial electron donor, which in vitro directly donates the electrons to the crARC Mo centre without the need of its protein partners (Figure 1). Nevertheless, the NADH donates the electrons to crCytb5-R, which afterward go to crCytb5-1 and then to the crARC Mo centre where the NHC reduction takes place, as shown in Figure 1. The use of two different electron donors allows a better understanding of the reason why crARC loses its activity. If crARC loses its dithionite-dependent activity, it will be considered that its Mo Cofactor centre is not working properly. However, if crARC only loses its NADH dependent activity, it is because crARC does not have a proper interaction with its partners, crCytb5-1 and crCytb5-R.

**Figure 2:**
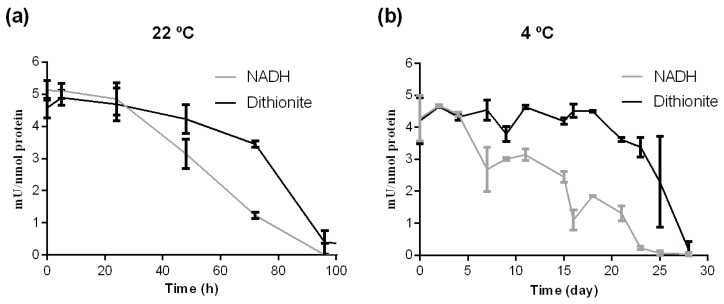
The crARC thermal and temporal stability. The crARC protein was incubated at (**a**) 22 °C or at (**b**) 4 °C for the indicated times and then the crARC activity was assayed in vitro using benzamidoxime as a substrate and either dithionite or NADH plus crCytb5-1 and crCytb5-R as electrons donors. The detection limit was 0.06 mU/nmol.

As shown in Figure 2a after incubation at 22 °C, crARC showed higher activity with dithionite than with NADH. The crARC activity measured with NADH after 72 h of incubation at 22 °C was only around 20% of the initial activity. However, under the same condition, this activity with dithionite was still 80% of the initial value. This means that the crARC stability at 22 °C was very high, since in both cases, its activity took several hours, around 100, to disappear completely.

As shown in Figure 2b, when crARC was incubated at 4 °C, its activity with dithionite was always from 2 to 5 times higher than with NADH. At 4 °C, the NADH-dependent crARC activity disappeared after 23 days; however, with dithionite, 80% of its initial activity was still observed. The crARC activity with dithionite took around 28 days to disappear. From these data, it can be concluded that the crARC protein protects the Mo Cofactor bound to its active centre very efficiently. Generally, the half-lives of the activity of the main known Mo enzymes are very short at room temperatures [23]. Therefore, in comparison to the other Mo enzymes, crARC seems to be different in this aspect.

### 2.2. The Interaction of crARC, crCytb5-1 and crCytb5-R

As shown previously, in vivo crARC needs two other proteins, crCytb5-1 and crCytb5-R, to carry out NHC reduction from NADH [17]. The highest reductase activity was obtained with an enzyme system reconstituted in vitro at a ratio of approximately 1:1:0.1 (crARC:crCytb5-1:crCytb5-R) with the human and Chlamydomonas system [15,17]. We performed a series of experiments to verify if there is indeed a real protein complex between these three proteins, to know its stoichiometry and whether or not it coincides with the obtained for the enzyme system reconstituted. We studied the interactions of these three proteins by size exclusion chromatography (SEC). SEC is a simple and mild chromatographic technique that separates molecules on the basis of size differences. In addition, a strong enough interaction among the proteins studied is needed to be maintained during the course of the SEC, which would result in changes of the chromatographic profiles of the protein mix with respect to those of separate proteins. The high crARC temporal stability found previously allowed us to perform the SEC with the confidence of no protein degradation until the end of experiment.

**Figure 3:**
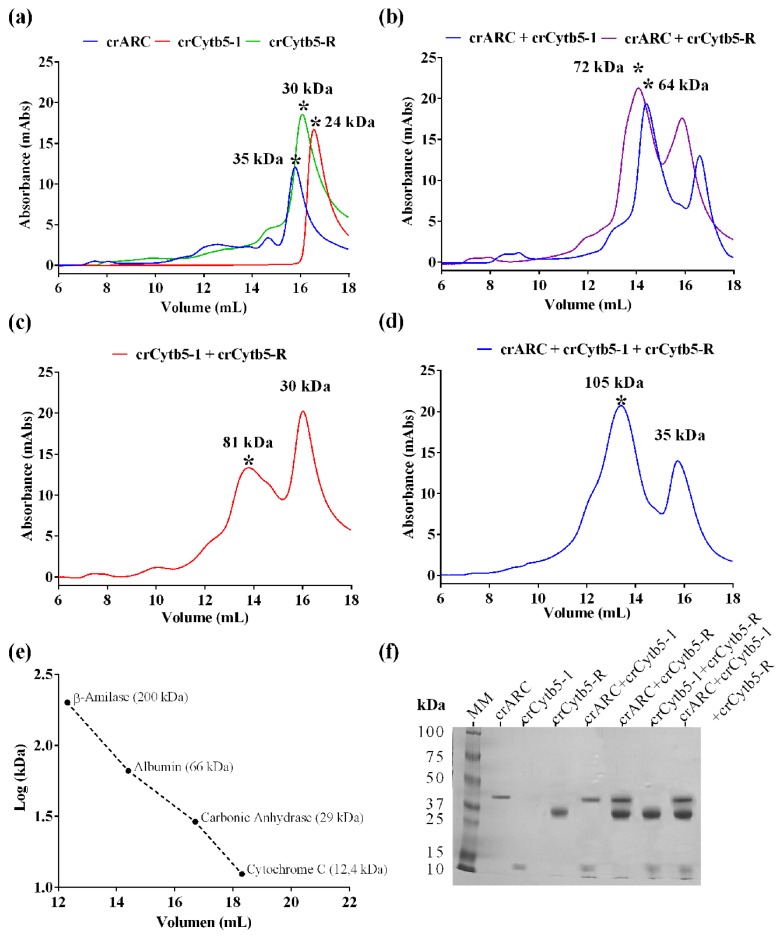
Interaction studies between crARC, crCytb5-1 and crCytb5-R. The SEC profiles of the indicated samples are shown. In these experiments, the proteins crARC, crCytb5-1 and crCytb5-R were loaded separately and then together in different combinations to observe the appearance of new peaks. (**a**) The proteins loaded separately; (**b**) crARC, with crCytb5-1, and crARC with crCytb5-R; (**c**) The crCytb5-1 protein with crCytb5-R; (**d**) The proteins crARC plus, crCytb5-1 and crCytb5-R together; (**e**) Calibration curve obtained using the *M*w protein marker; the graph represents the *M*w versus the logarithm of the elution volume. The absorbance was measured at 280 nm; (**f**) sodium dodecyl sulfate (SDS)-polyacrylamide gel electrophoresis of the protein peak indicated with the symbol * obtained in the SEC.

The proteins crARC, crCytb5-1 and crCytb5-R were incubated either alone or together for 10 min and then the samples were loaded on a SEC column. If during the incubation period, any kind of protein complex is formed, the elution profile will change with respect to those of proteins loaded alone. The molecular weight (*M*_W_) of eluted proteins or complex will be estimated by comparing with protein markers of known *M*_W_ loaded under the same conditions (Figure 3e).

It is important to note that for some proteins, the *M*_W_ estimated by SEC are larger than those predicted from their amino acids sequence. This means that the *M*_W_ obtained by SEC can be overestimated. This overestimation happens because the proteins have to pass through the pores of the column in a spherical way and the remaining volume to complete the spherical shape is added [24]. That is not the case of the proteins markers used, which are close to spheres. However, underestimation of molecular weight by SEC is conceivable as well [25].

Figure 3a shows the SEC profiles of the proteins crARC, crCytb5-R and crCytb5-1 when they were loaded alone. The estimated *M*w of the main peak obtained with crARC was 35 kDa (Figure 3a, in blue), which is very close to 35.1 kDa, the predicted *M*w of one crARC monomer. This indicates that in the absence of its partners, crARC occurs mostly as a monomer, as reported for the N*-*terminal truncated homologue hmARC1 [26]. The estimated *M*w of the crCytb5-1 peak was 24 kDa (Figure 3a, in red). The predicted *M*w of one crCytb5-1 was 12.7 kDa, which indicates that crCytb5-1 occurs as a dimer in the absence of its partner.

The estimated *M*w for crCytb5-R alone was 30 kDa (Figure 3a, in green), which is close to its predicted value, 28.7 kDa, suggesting that this protein occurs as a monomer in the absence of partners. The crARC and crCytb5-1 incubated together showed a main peak with an estimated *M*w of 64 kDa (Figure 3b, in blue), which is compatible with the complex of 1 crARC and 2 crCytb5-1 (predicted *M*w of 60.5 kDa). The other peak that appears has an estimated *M*w of 24 kDa and probably corresponds to an excess of crCytb5-1 dimers (Figure 3b).

The SEC of crARC and crCytb5-R incubated together gave a peak with an estimated *M*w of 72 kDa (Figure 3b, in purple), compatible with a complex of 1 crARC and 1 crCytb5-R (predicted *M*w of 63.8 kDa). Also, a minor peak of 30 kDa appeared that probably corresponds to an excess of crCytb5-R monomers (Figure 3a). When crCytb5-R and crCytb5-1 were incubated together after the SEC, two peaks were observed with an estimated *M*w of 81 kDa for the wider and 30 kDa for the thinner one (Figure 3c). The wider peak corresponds to a complex of 1 crCytb5-R and 4 crCytb5-1 (predicted *M*w of 79.8 kDa), and the thinner one corresponds to an excess of crCytb5-R monomers.

When the three proteins (crARC + crCytb5-1 + crCytb5-R) were incubated together, after the SEC, the chromatogram showed a main peak with an estimated *M*w of 105 kDa and one smaller one with *M*w of 35 kDa (Figure 3d). The minor peak of 35 kDa would correspond to an excess of crARC monomers. It is remarkable that this 105 kDa peak only appeared when the three proteins were incubated together. Therefore, it was probably formed due to some kind of interaction among the three proteins. The 105 kDa complex is compatible with a composition of 1:2:1 (predicted Mw of 89.2 kDa), 1:3:1 (predicted *M*w of 101.9 kDa) or 1:1:2 (predicted *M*w of 105.2 kDa) for crARC-crCytb5-1-crCytb5-R. Other possible stoichiometries like 1:2:2, or 2:1:1 have been rejected because their predicted *M*w are considerably higher than 105 kDa.

To determine whether the SEC peaks were formed by the proposed protein complexes, the fractions of the main peaks in each chromatographic profiles were loaded on a polyacrylamide gel for electrophoresis. The analyzed peaks are those marked with an asterisk in Figure 3. As shown in Figure 3f, all the protein bands that appeared in the gel are in agreement with the proposed interactions. As can be seen in the gel electrophoresis, the 105 kDa peak is related to the complex of the three proteins, crARC + crCytb5-1 + crCytb5-R (Figure 3f).

To study if this three-protein complex is catalytically active after SEC, we measured its benzamidoxime reduction activity with just NADH and without adding any additional proteins partners. An activity of 0.547 ± 0.082 mU/nmol protein was obtained. The benzamidoxime reduction of the mixture before its loading to the SEC column was 0.861 ± 0.129 mU/nmol protein. This means that this complex retained 63% of its activity after SEC. Therefore, in this complex, the three proteins are assembling properly to perform the catalysis and retain its enzymatic activity.

### 2.3. Study of Different crARC Variants

**Figure 4:**
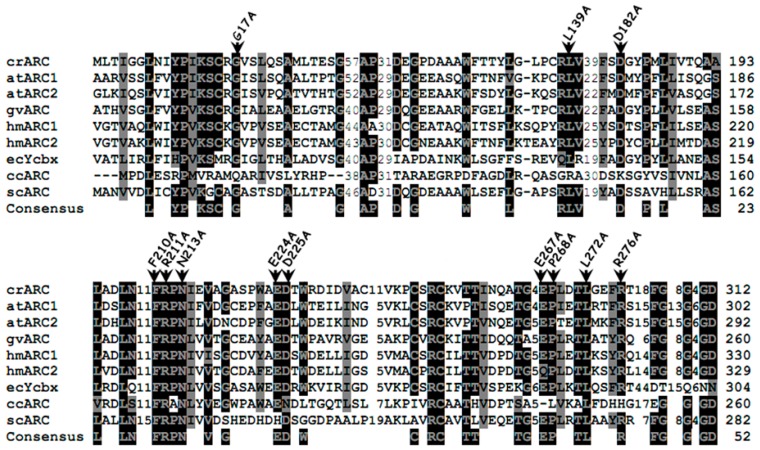
Multiple-sequence alignment of different mARC proteins. The sequences and accession numbers (shown into parentheses; GenPept accession numbers begin with XP and NP; others are proteins deduced from GenBank sequences) are as follows: crARC, *Chlamydomonas reinhardtii* mARC (XP_001694549); atARC1, *Arabidopsis thaliana* mARC1 (NP_174376); atARC2, *Arabidopsis thaliana* mARC2 (NP_199285); gvARC *Gloeobacter violaceus* PCC 7421 mARC (NP_926027); hmARC1, human mARC1 (*Homo sapiens*) (NP_073583); hmARC2, human mARC2 (NP_060368); YcbX, *Escherichia coli* YcbX (NP_415467); ccARC, *Caulobacter crescentus* mARC (AAK22857); and scARC, *Streptomyces coelicolor* A3ARC (CAC04053). The positions of residues mutated to alanine in crARC are indicated by the black arrowheads. Highly conserved amino acids are shown on a black background, and moderately conserved amino acids are shown on a gray background. The consensus sequences have been calculated with a threshold of 75% with the BioEdit v.7.0.9 program (Reprinted with permission from [17]).

Several crARC point mutant variants in different conserved amino acids were constructed and their behaviors were compared with the wild type protein afterwards. Figure 4 shows the alignment of crARC with other mARC from organisms of different kingdoms where different conserved amino acids are highlighted. We have generated 12 different variants exchanging the indicated amino acids for alanine. Once obtained, the recombinant proteins were over expressed and purified in *E. coli* (*Escherichia coli*). The mutant P268A was discarded because of the low yield obtained in its purification, probably because the protein appeared in bacterial inclusion bodies. The remaining 11 crARC variants were purified to proceed further with their characterization and analysis.

**Figure 5:**
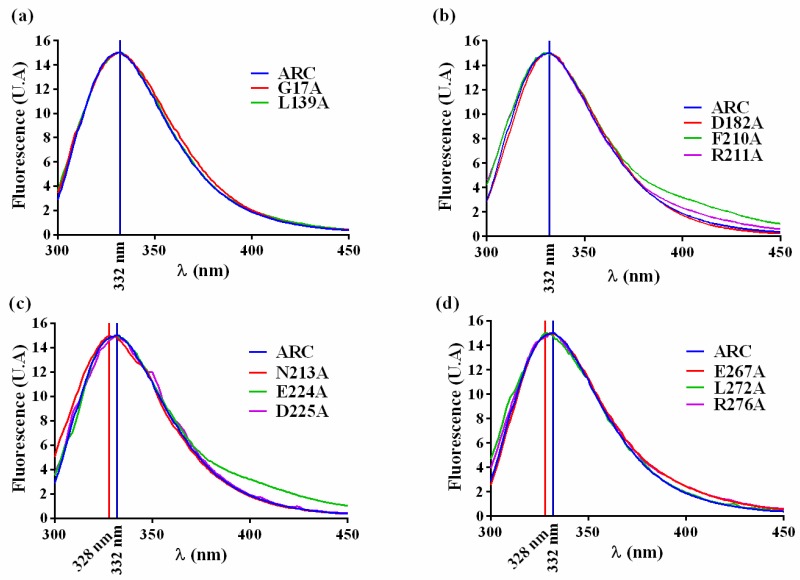
The fluorescence spectrums of crARC wild type and variants (**a**–**d**).

The substituted amino acid should not perturb the overall folding of the crARC protein, and if it happens, the mutant should be discarded. Therefore, to find out whether the overall tree dimensional (3D) crARC structure of these 11 mutants had changed compared with the wild type, the fluorescence spectrum of aromatic amino acids of those proteins was compared to that of the wild type. As crARC has 10 tryptophans distributed along its sequence, if any of the substitutions affect the folding of the protein, its fluorescence spectrum will be altered. Therefore, the maximum of fluorescence and the spectrum shape of the crARC variants have to be the same as the wild type crARC; otherwise, it indicates that the overall 3D structure of the variants has been affected by changing the amino acid. After the analysis, the 8 crARC variants G17A, L139A, D182A, F210A, R211A, E224A, E267A and R276A had fluorescence spectra very similar to the wild type protein crARC (Figure 5). However, 3 variants, N213A, D225A, and L272A had notable differences compared with the wild type, crARC. In these 3 variants, the maximum peak appeared at different wavelength compared with the wild type, 328 nm instead of 332 nm, and showed shoulders in their spectrum different from the wild type (Figure 5). This indicates that the overall 3D structures of these 3 variants are different from the wild type crARC protein, probably because the changed amino acids were involved in maintaining the overall 3D crARC structure. Therefore, these 3 variants were rejected for further analysis.

### 2.4. The Reduction Activity of the crARC Variants

**Figure 6:**
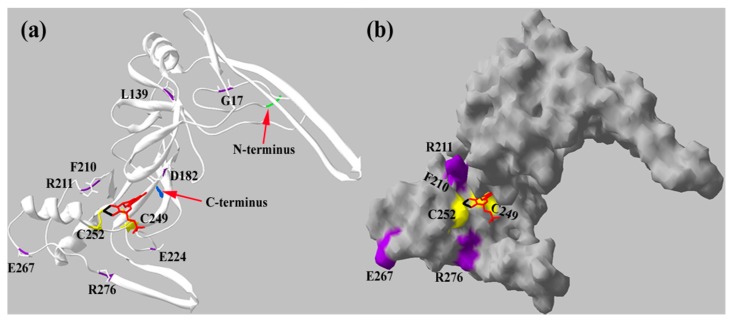
Predicted in silico crARC protein structure. The structures were obtained with Swiss-PdbViewer and Swiss-model programs in (**a**) secondary structure and in (**b**) protein surface. This structure was made in two parts, because the part with the β-barrel domain presents an identity of 32.2% with the BoR11 protein (2exn.1.A); on the other hand, the second part (MOSC domain) presents an identity of 14.88% with the yuaD protein (1oru.1.A). The N-terminus is in green, and the C terminus in blue, the Mo Cofactor is in red, in black is the Mo, in yellow are the cysteines 249 and 252 connecting the Mo Cofactor and in purple are the mutated residues.

Thus far, the crystallographic 3D structure of mARC has not been resolved. Therefore, we observed in silico predicted structure of crARC (Figure 6) to locate the positions of the 8 amino acids, G17, L139, D182, F210, R211, E224, E267 and R276. In this predicted structure, we have also located the two amino acids, C252 and C249, which previously showed involvement in the Mo Cofactor binding to crARC [17].

**Figure 7:**
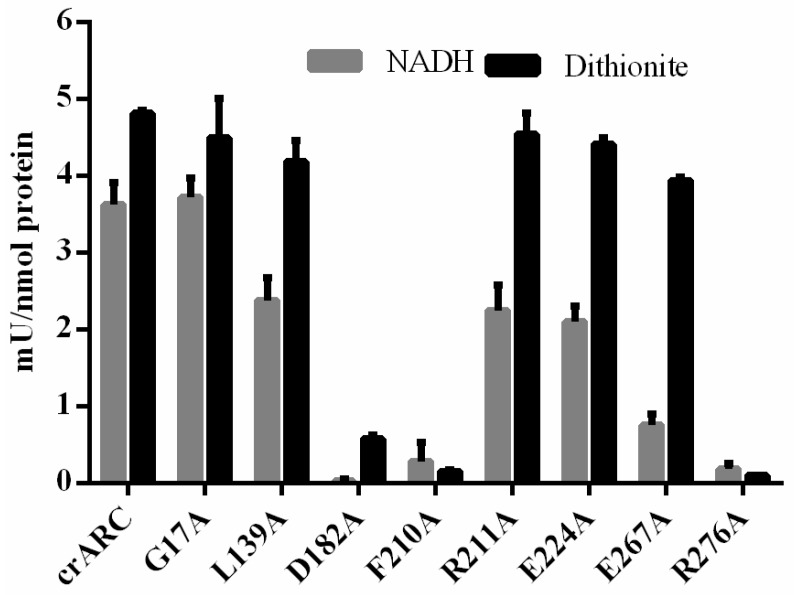
The reduction activity of crARC wild-type and variants. The crARC HAP reduction activity was determined with dithionite (grey bars) or with crCytb5-1/crCytb5-R plus NADH (black bars). The detection limit was 0.045 mU/nmol.

A good indication of the role of each studied amino acid in the crARC function is to know their effect on the reduction activity compared with the wild type. As shown in Figure 2, the crARC activity was determined in two ways, either using dithionite or using crCytb5-1 and crCytb5-R with NADH as electron donors. The results of these experiments (Figure 7) showed that the mutants, D182A, F210A and R276A almost completely lost the mARC activity with dithionite. Since dithionite donates the electrons directly to the crARC Mo Cofactor centre (see Figure 1), these residues might play a role in the suitable Mo Cofactor binding to crARC. As shown in Figure 6, the amino acids D182 and the F210 are in close proximity to the Mo Cofactor centre, which agrees with our hypothesis. However, the amino acid R276 is not in close proximity to the Mo Cofactor, and it seems not to be directly involved in the Mo Cofactor chelation. Therefore, R276 could be more strongly involved in a proper Mo Cofactor binding pocket formation. The crARC activities tested with dithionite of the remaining mutants, G17A, L139A, R211A, E224A and E267A were very similar to the wild type, which would indicate that these amino acids are not necessarily related to the Mo Cofactor chelation or the binding pocket formation. On the other hand, we also measured the crARC activity with NADH, which as mentioned before needs crCytb5-1 plus crCytb5-R. Consistently, the variants, D182, F210 and R276, which did not have dithionite dependent activity, do not have NADH dependent activity as well, thus reinforcing the proposal that in these variants, the Mo Cofactor centre does not work properly.

In the 5 other variants, G17A, L139A, R211A, E224A and E267A, the NADH crARC activity tested was considerably lower than in the wild type except for G17A, which was very similar to the wild type. The variant E267A was the one with lowest NADH-dependent activity, i.e., only about 25% of the wild type activity. The NADH crARC reduction activity of the variants L139A, R211A and E224A was between 50%–60% of the wild type. These data indicate that the amino acids, E267, L139, R211 and E224 are important but not essential for the correct electron transfer from the NADH to the crARC Mo Cofactor centre. These data suggest that these 4 amino acids are involved in the interaction between crARC and its partners, crCytb5-1 and crCytb5-R.

### 2.5. The Mo Cofactor Content of the crARC Variants

**Figure 8:**
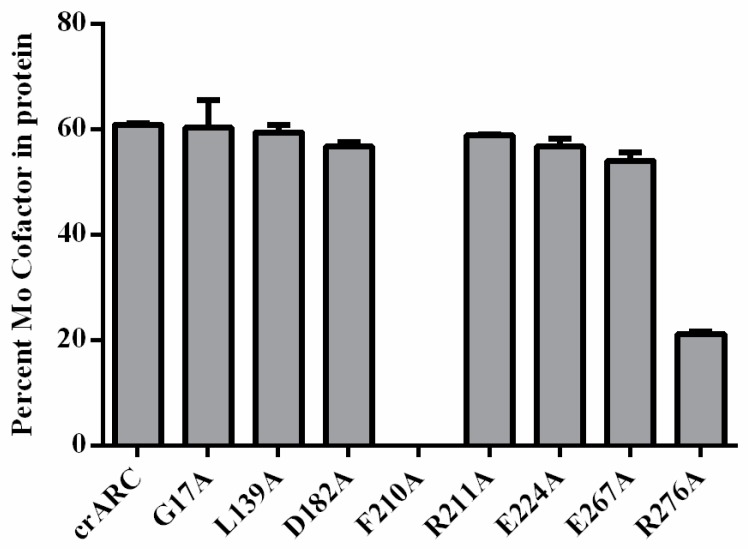
The Mo cofactor content in the crARC wild type and variants. The content was measured by the Form A method in a HPLC with a fluorescence detector.

The amount of Mo Cofactor in the crARC variants was measured by high performance liquid chromatography (HPLC) using the Form A method. As shown in Figure 8, the mutants, G17A, L139A, D182A, R211A, E224A, and E267A showed very similar Mo Cofactor content as the wild type. It is very interesting that the variant D182A lost its reduction capacity with both dithionite and NADH (Figure 7); however, it contains the Mo Cofactor, which suggests two possibilities: (i) the Mo Cofactor bound to the variant D182A could not be in the proper form to accept the electrons form dithionite or from NADH; and (ii) the Mo Cofactor bound to the D182 variant could accept the electrons but was not able to donate them to the substrate, perhaps because the substrate could not bind properly. The variant R276A also lost its reduction capacity with dithionite and NADH (Figure 7), but still retained 30% of the Mo Cofactor content (Figure 8). This indicates that as well as in the variant D182A, the Mo Cofactor bound is not in the proper form to accept the electrons or to donate them to the substrate. Interestingly, the variant F210A was the only one studied with no Mo Cofactor bound at all. This indicates that the F210 is essential for Mo Cofactor binding, which explains the reason of not having crARC reduction activity. In summary, D182, F210 and R276 are necessary for mARC enzymatic activity; and for the Mo Cofactor chelation, R276 is important while F210 is essential.

### 2.6. The Oligomerization of the crARC Variants

**Figure 9:**
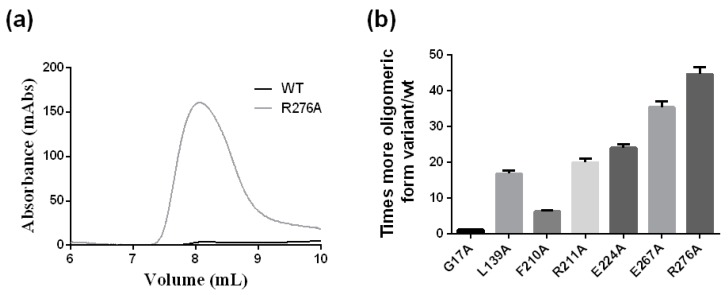
The oligomerization of the crARC variants. This study was conducted by chromatography in a SEC column loading the wild and variants separately. (**a**) Chromatographic profiles of wild type crARC and the mutant R276 variant; (**b**) Oligomeric states of the crARC variants with respect to the wild type.

The chromatographic behavior in a SEC column of the crARC variants was also studied. Interestingly an oligomeric state has been observed in the human homolog hmARC1 [27]. Therefore, the oligomerization degree was studied to distinguish whether they were all monomers, just like the wild type (Figure 3a), or they might be some oligomers. Therefore, the wild type and the different crARC variants were loaded in a SEC column, and their chromatographic profiles were compared. As shown in Figure 3a the wild type crARC protein mostly behaves as a monomer with almost no peak eluting at around 8 mL. Interestingly, in some of the variants, a huge peak appeared at the volume of 8 mL. This peak can be observed in Figure 9a that shows the chromatographic profile of the wild type versus the variant R276A. The volume at which this peak appeared indicates an *M*w of 1100 kDa, which corresponds to about 30 crARC units. Figure 9b represents the amount of oligomeric forms that appears in each of the mutants studied. The variants G17A and W128A look very similar to the wild protein without almost any oligomeric form. The variants with a higher percentage of the oligomeric state were E267A and R276A with around 40–50 times more aggregated than the wild type protein.

The variant R276A has no crARC reduction activity (Figure 7); however, the dithionite-dependent crARC reduction activity of the E267A was similar to the wild type. Also, mutants with similar reduction activity like G17A and E224A are monomers and oligomers respectively. These data indicate that the oligomerization state is not related to the crARC reduction activity.

The variants E224A, R211A and L139A have around 20–30 times higher oligomeric states than the wild type protein. Figure 7 shows that the crARC reduction activities of these mutants are very similar to the wild type, which supports the previous hypothesis that the oligomerization state has no influence on the crARC reduction activity.

The variant F210A shows around 10 times more oligomeric forms than the wild type and has no Mo Cofactor bound at all. Variants with approximately the same amount of Mo Cofactor have very different amounts of crARC oligomers compared with G17A and E267. These data indicate that the amount of Mo Cofactor is not the reason why some crARC variants aggregate in oligomers.

## 3. Discussion

In this study, we have addressed several important points that have not yet been studied in any protein of the mARC family. Free Mo Cofactor is a very unstable molecule that loses Mo above pH 7.6 under aerobic conditions [28] and has a half-life of around 1 h depending on the components in the buffer solution [29]. Therefore, under aerobic conditions the Mo Cofactor in Chlamydomonas binds to a carrier protein (MCP) that is involved in the transfer, storage, protection and insertion of the Mo Cofactor in the apo-enzyme [22]. Under aerobic conditions, the half-life of the Mo Cofactor bound to the MCP at 4 °C is around 3 days [23], and as shown in Figure 2b at 4 °C, the crARC activity has a half-life of around 20 days and 15 days with dithionite and NADH, respectively. At 22 °C, the half-life of the Mo Cofactor bound to the MCP is around 24 h [23], and 70 h and 55 h for dithionite and NADH dependent crARC activity, respectively. Thus, crARC is able to protect its bound Mo Cofactor for at least as long as the MCP at 22 °C and even more at 4 °C. Therefore, under aerobic conditions, crARC is a very stable Mo enzyme compared with other Mo enzymes like NR [30]. Human mARC, in addition to its reduction capacity, has been shown to donate Mo Cofactor to Mo-deficient NR [9]. This fact, together with the high stability observed in crARC, suggests that crARC might also play a role, under some conditions, in Mo Cofactor storage or as an insertional protein, buffering the amount of Mo Cofactor inside the cells. However, this hypothesis needs more future research.

The stoichiometry of the interaction between crARC, crCytb5-1 and crCytb5-R could be 1:2:1, 1:3:1 or 1:1:2 respectively, (Figure 3d); future experiments will be needed to clarify in more detail which of them is correct. None of these stoichiometries agrees with 1:1:0.1, which has been described in Chlamydomonas and humans as the one that produce the highest reductase activity with the enzyme system reconstituted in vitro [15,17]. It has to be pointed out, that the 1:1:0.1 estimation reflects optimal conditions of the in vitro reductase assay, while here we have measured and detected the formation of a protein complex. It has to be noted that the 105 kDa complex is catalytically active and able to perform the NHC reduction just by adding NADH to the complex, suggesting that this in vitro complex is not an artifact. We do not yet know the reason for the different stoichiometry between the protein complex and the reduction system reconstituted in vitro. Therefore, we propose that different stoichiometric behaviors depending of the organism or the in vivo physiological state may be possible.

The subcellular localization of mARC proteins is not well defined. The mammalian Mo enzyme is located on the outer mitochondrial membrane. [4,27,31]. Moreover, a location on the inner mitochondrial membrane [32] as well as peroxisomal location [33] of the Mo is reported as well. However, NHC reductase activity in humans is strictly dependent on cytochrome *b*_5_ reductase CYB5R3 and mitochondrial cytochrome *b*_5_ CYTB5 [14,15]. The latter enzyme seems to be located solely on the outer membrane [34]. Thus, mammalian ARCO reductase activity is likely exclusively associated with this submitochondrial compartiment. However, the Arabidopsis and Chlamydomonas counterparts lack a clear targeting signal for organelle export [3]. This suggests that they could be free in the cytoplasm. The stoichiometries found among crARC and its partners were obtained in vitro using purified proteins, but the fact that some mARC proteins are attached to different subcellular membranes, could change the real in vivo stoichiometry. However, we think that the in vitro stoichiometries found for the studied proteins are a good first indication of what may be happening in vivo, though future experiments on this aspect are needed.

From the study of 12 different crARC mutant variants, it was deduced that three amino acids from crARC, D182, F210 and R276 are essential for NHC reduction (Figure 7), since their mutation to alanine completely abolished the crARC reduction activity. These mutants might have a deficient or an incorrect Mo Cofactor binding. We have also identified 4 amino acids E267, L139, R211 and E224, which are important but not essential for the correct electron flow from NADH to crARC. The reason may be that these amino acids are involved in the interaction with crCytb5-R or crCytb5-1. However with the current data, we cannot discriminate which ones of these alternatives would be the correct one. Interestingly, crARC seems to have basic amino acids in the entire outer part where the Mo Cofactor is bound, and it has been described that there are mostly acid amino acids in the outside of crCitb5-1 and crCitb5-R [35], so this would explain in part the interaction between these three proteins.

We have also identified that two amino acids, F210 and R276 (Figure 8), are important for Mo Cofactor binding. The variant R276A had around 30% of the wild type Mo Cofactor content, which suggests that this residue is notably important but not essential for Mo Cofactor binding. However, amino acid R276 is not in close proximity to the Mo Cofactor, similar to F210. Therefore, R276 could not be directly involved in Mo Cofactor chelation; instead, it could be involved in a proper Mo cofactor binding pocket formation. Therefore, we propose that R276 might be involved in proper Mo Cofactor accommodation in the crARC active centre. Notwithstanding, F210 is essential since its exchange completely abolished the Mo Cofactor binding. In previous experiments, we detected that C252 is also essential for Mo Cofactor binding [17]. The predicted in silico crARC structure (Figure 6) shows that F210 is in close proximity to where the Mo Cofactor is supposed to bind. This work has highlighted F210, as previously found for C252, to be a critical residue for Mo Cofactor binding.

The mARC proteins contain two conserved domains: an N-terminal β-barrel domain and a C-terminal MOSC domain [5]. The mARC proteins show several conserved patches of hydrophobic residues and the absolutely conserved cysteine located in the C-terminus, which could be considered part of their signature [17]. The N-terminal β-barrel domain, which in standalone form is undetectable in other proteins, is predicted to build a β sheet-rich fold like structure. This particular domain may have specific roles in interaction with substrates of these enzymes. In the predicted mARC structure (Figure 6), it can be observed that from A66 to P79, there is a β sheet with the hydrophobic charge exposed to the outside, which possibly can be used to interact with a membrane as suggested in the human mARC [27]. The mARC β sheet has also been involved in the origin of the aggregates formation occurring in the human mARC [27]. However, we have not mutated the amino acids of this domain in crARC so it is unknown whether this β sheet is also involved in the aggregate formation in Chlamydomonas. The mammalian SO is another Mo enzyme where a high degree of oligomeric forms has been detected [36] probably due to the lack of the heme domain in this protein, with the subsequent exposure of hydrophobic surface patches.

In crARC variants, the oligomeric state increases as the amino acid that is changed is closer to the C-terminus (Figure 9b). A higher percentage oligomeric state occurs in E267A and R276A, which are close to the C-termini. This could indicate that in crARC, the C-terminus may be involved in protein aggregation to form the oligomers, because its alteration increases the oligomeric forms observed. Our data also indicate that the degree of oligomerization is not related to the crARC reduction activity or to the Mo Cofactor binding capacity.

In spite of the role that the mARC enzyme plays in prodrug activation, its exact biological function remains elusive. mARC is able to reduce very different kinds of substrates, not all of them NHC, like nitrite [18] and can use different kinds of partners, like NR [19]. ARCO is assumed to be involved in the detoxification reactions of mutagenic and toxic NHCs like *N*-hydroxylated nucleobases and nucleoside [9,10], as well as aromatic hydroxylamines [37].

In addition, in vitro studies have shown that human mARC is also able to catalyze the reduction of the NO precursor NOHA to arginine [20]. A variety of several enzymes has been involved in NO synthesis by reducing nitrite, and several of them are Mo enzymes [21]. It has been recently shown that human mARC, apart from the NHC reductions, is also able to catalyse the reduction of nitrite to NO using crCytb5-1 and crCytb5-R as partners under anaerobic conditions [18]. In Chlamydomonas, we have described that the Mo enzyme NR is able to replace the function of crCytb5-R and crCytb5-1 and it can transfer the electrons from NAD(P)H to crARC, which synthesizes NO from nitrite [19]. It is also interesting to note, that various investigations identified linkages to energy metabolism [38], notably diabetic mellitus [39] and lipid synthesis [31]. Interestingly the down-regulation of the human mARC resulted in a significant decrease of the intracellular lipid levels [31]. However, the exact role of mARC in these fields like lipid synthesis will require further research.

## 4. Materials and Methods

### 4.1. Chemicals

HAP was purchased from ICN Biochemicals (Irvine, CA, USA). The other chemicals were purchased from Sigma-Aldrich (Madrid, Spain).

### 4.2. Bacterial Strains and Culture Conditions

*The E. coli* strains were grown on LB (Luria-Bertani) medium. The *E. coli* strain TP1000 (*mobA*) [40] was used for the expression of crARC because it accumulates eukaryotic Mo Cofactor. The *E. coli* strain BL21 (DE3) [41] was used for the expression of crCytb5-1 and crCytb5-R.

### 4.3. Cloning of cDNA for Recombinant Protein Expression.

The generation of the cysteine to alanine mutants of crARC was performed by PCR mutagenesis [42]; the primers used are shown in Table S1 (Online Resource 1) and obtained cDNAs were cloned in pQE80, which allows the expression of N-terminal six Histidines tagged fusion proteins in *E. coli* (Qiagen, Hilden, Germany).

### 4.4. Expression and Purification of Recombinant Proteins

Standard expression of the crARC and crARC cysteine to alanine mutants was performed in freshly transformed *E. coli* TP1000. Cells were grown aerobically in LB medium to an OD_550_ equal to 0.1 before induction. TP1000 cells were induced with 10 µM isopropyl-β-d-thiogalactopyranoside (IPTG) and additionally supplemented with 0.1 mM sodium molybdate to initiate recombinant expression. BL21 cells were induced with 100 µM IPTG for expression. crCyb5-1 and crCytb5-R were purified as described previously [17]. Cells expressing proteins with heme groups were supplemented with 1 mM aminolevulinic acid to support heme synthesis. After induction, the cells were grown for 36 h at 22 °C. Purification of recombinant proteins was performed by the Ni-nitrilotriacetic acid (Ni-NTA) matrix, as recommended by the supplier (Qiagen), under native conditions at 4 °C, using minimal volumes of washing buffers to reduce dissociation of bound Mo from the proteins. The protein fractions were analyzed by SDS-polyacrylamide gel electrophoresis and only the pure fractions were taken and immediately desalted on a PD10 gel filtration column previously equilibrated with 100 mM Tris-HCl, pH 7.2. Protein concentration was determined by UV absorption measurements using the calculated extinction coefficient [43] of the analyzed polypeptides.

### 4.5. DNA Sequencing and Sequence Analysis

The DNA sequencing and sequence analysis were performed as described previously [17].

### 4.6. HAP and Adenine Quantification

HAP and adenine were separated and quantified by HPLC as described previously [17].

### 4.7. Benzamidine and Benzamidoxime Quantification

Benzamidoxime and Benzamidine were separated and quantified by HPLC as described previously [26]. The HPLC analysis was performed on an Agilent series 1200 from Agilent Technologies (Santa Clara, CA, USA).

### 4.8. The Quantification of the crARC Reduction Activity

The HAP or Benzamidoxime reduction by crARC was determined as described previously [19] with minor modifications. Incubations were carried out under aerobic conditions at 37 °C in a shaking water bath. For the enzyme system reconstituted in vitro for the crARC NADH-dependent activity, standard incubation mixtures in a volume of 150 µL contained 100 pmol crARC, 10 pmol crCyt b5-R, 100 pmol crCytb5-1, 1.0 mM NADH and 0.5 mM HAP or 1 mM of benzamidoxime and 100 mM potassium phosphate buffer at pH 6.5. After pre-incubating for 3 min at 37 °C, the reaction was started by adding NADH, then terminated after 15 min by adding 150 µL of methanol. For the crARC dithionite-dependent activity, standard incubation mixtures of the reconstituted system contained crARC 100 pmol, 0.5 mM HAP or 1 mM benzamidoxime, 2 mM benzylviologen and 3 mM dithionite in 100 mM potassium phosphate buffer at pH 7.4. After 3 min of pre-incubation at 37 °C, the reaction was started by adding dithionite and terminated after 15 min by adding 15% methanol. After centrifugation, the supernatant was analyzed by HPLC. The protein mixture used for the SEC was 15 pmol crARC, 15 pmol crCyt b5-R, 15 pmol crCytb5-1, and the crARC reduction activity measure in the same way as for the enzyme system reconstituted in vitro. One unit of crARC activity is defined as the amount of enzyme causing the production of 1 µmol of adenine or benzamidine per minute under the described conditions.

### 4.9. Determination of the Organic Motive of Mo Cofactor

To measure the amounts of Mo Cofactor bound to the proteins, its organic motif (MPT) was analyzed by the Form A method, performed as reported [44].

### 4.10. Molecular Weight Determination by SEC

To determinate the *M*w of native protein or the protein complex, the AKTA STAR chromatographic system was used with the SEC column “Superdex 200 10/300 GL” from GE Healthcare (Barcelona, Spain). The proteins amounts used for the SEC were 15 pmol for each one in the mixture. The chromatographic method was an isocratic phase with 50 mM of phosphate buffer at pH 7.2, 0.15 M of NaCl and a flow rate of 0.75 mL/min. After the SEC, the fractions were collected and immediately desalted on a PD10 gel filtration column previously equilibrated with 100 mM Tris-HCl, pH 7.2. Using *M*w markers proteins, a calibration curve was obtained representing the *M*w versus the logarithm of elution volume to obtain the *M*w of any protein using its elution volume obtained after the SEC by extrapolation. After the SEC, the protein amount was measure by the Bradford method [45].

### 4.11. Studies of Tertiary and Quaternary Structure of crARC Mutants

A fluorescence technique was used to determine whether the obtained crARC variants have the same basic structure as the crARC wild protein. This technique measures the fluorescence emission by the excitation of tyrosine, phenylalanine and tryptophan amino acids; tryptophans result in high fluorescence emission [46]. With changing the environment of these amino acids, the fluorescence emission varied. The crARC protein has 10 tryptophans in its 330 amino acids; therefore, it can be well studied in environments with these 10 amino acids and if it has changed with respect to the wild type.

### 4.12. Software Used to Predict the Three-Dimensional Structure of crARC

The software used was the Swiss-PdbViewer Program of tertiary structures and three-dimensional alignments of already crystallized structures online version, http://spdbv.vital-it.ch/Swiss-Model. In the program, prediction of protein structures from their primary structure is based on searching crystallized proteins with similar sequences, entropies and enthalpies; the most favorable and crystallized protein-like structures are sought. The program outputs a .pdb file that we can view using the Swiss-PdbViewer, http://swissmodel.expasy.org/AutoDockTools-1.5.6 was used to insert the Mo Cofactor into crARC structure.

## 5. Conclusions

In conclusion, we have found that crARC activity is very stable over time. We have characterized a protein complex formed between crARC and its partners crCytb5-1 and crCytb5-R. In addition, the amino acids D182 and R276 are necessary in the NHC reduction activity, and F210 is essential in Mo Cofactor binding. We have also found that the alteration in the crARC C-terminus causes an oligomerization. Here, we have not addressed the crARC capacity to form NO from nitrite since it depends on NR as the crARC partner. Future experiments will be designed to address the stoichiometry and the role of the variants in the crARC-NR interaction for NO synthesis.

## Supplementary Materials

Supplementary materials can be found at www.mdpi.com/1422-0067/18/3/670/s1.
