# Supplementary material for: Study of Different Variants of Mo Enzyme crARC and the Interaction with Its Partners crCytb5-R and crCytb5-1
Source: Int J Mol Sci. 2017 Mar 21;18(3):670. doi: 10.3390/ijms18030670 (PMC5372681; doi:10.3390/ijms18030670)
Supplement: Supplementary file 1 [file ijms-18-00670-s001.pdf]

Table S1. List of primers used.

	Name	Primer 5'-3'	Mutant
1	CrARCG17Af	CATGTCGCGCTGTGTCCCTCC	**G17A**
	CrARCG17Ar	GAGGGACACAGCGCGACATGA	
2	CrARCL139Af	CCCTGTCGCGCGGTGCGCT	**L139A**
	CrARCL139Ar	AGCGCACCGCGCGACAGGG	
3	CrARCD182Af	CTTCAGCGCCGGCTACCCCA	**D182A**
	CrARCD182Ar	ATGGGGTAGCCGGCGCTGAAG	
4	CrARCF210Af	AACCGCGCCCGGCCCAACA	**F210A**
	CrARCF210Ar	TGTTGGGCCGGGCGCGGTT	
5	CrARCR211Af	AACCGCTTCGCGCCCAACA	**R211A**
	CrARCR211Ar	TGTTGGGCGCGAAGCGGTT	
6	CrARCN213Af	GCTTCCGGCCCGCCATTGAGGT	**N213A**
	CrARCN213Ar	ACCTCAATGGCGGGCCGGAAGC	
7	CrARCE224Af	CCTGGGCTGCGGACACCTGGC	**E224A**
	CrARCE224Ar	CGCCAGGTGTCCGCAGCCCAG	
8	CrARCD225Af	CCTGGGCTGAGGCCACCTGGC	**D225A**
	CrARCD225Ar	CGCCAGGTGGCCTCAGCCCAG	
9	CrARCE267Af	GGCGATGCGCCGCTGGACA	**E267A**
	CrARCE267Ar	TGTGTCCAGCGGCGCATCGC	
10	CrARCP268Af	GGCGATGAGGCGCTGGACA	**P268A**
	CrARCP268Ar	TGTGTCCAGCGCCTCATCGC	
11	CrARCL272Af	CTGGACACAGCGGGAGAGTTCAGGA	**L272A**
	CrARCL272Ar	GTCCTGAACTCTCCCGCTGTGTCCA	
12	CrARCR276Af	GAGAGTTCGCGACGGGCAAGGTG	**R276A**
	CrARCR276Ar	CACCTTGCCCGTCGCGAACTCTC	
